# Supplementary figures and images for: Egress-enhancing mutation reveals the inefficiency of non-enveloped virus cell exit
Source: PLoS Biol. 2025 Jun 24;23(6):e3003245. doi: 10.1371/journal.pbio.3003245 (PMC12212872; doi:10.1371/journal.pbio.3003245)

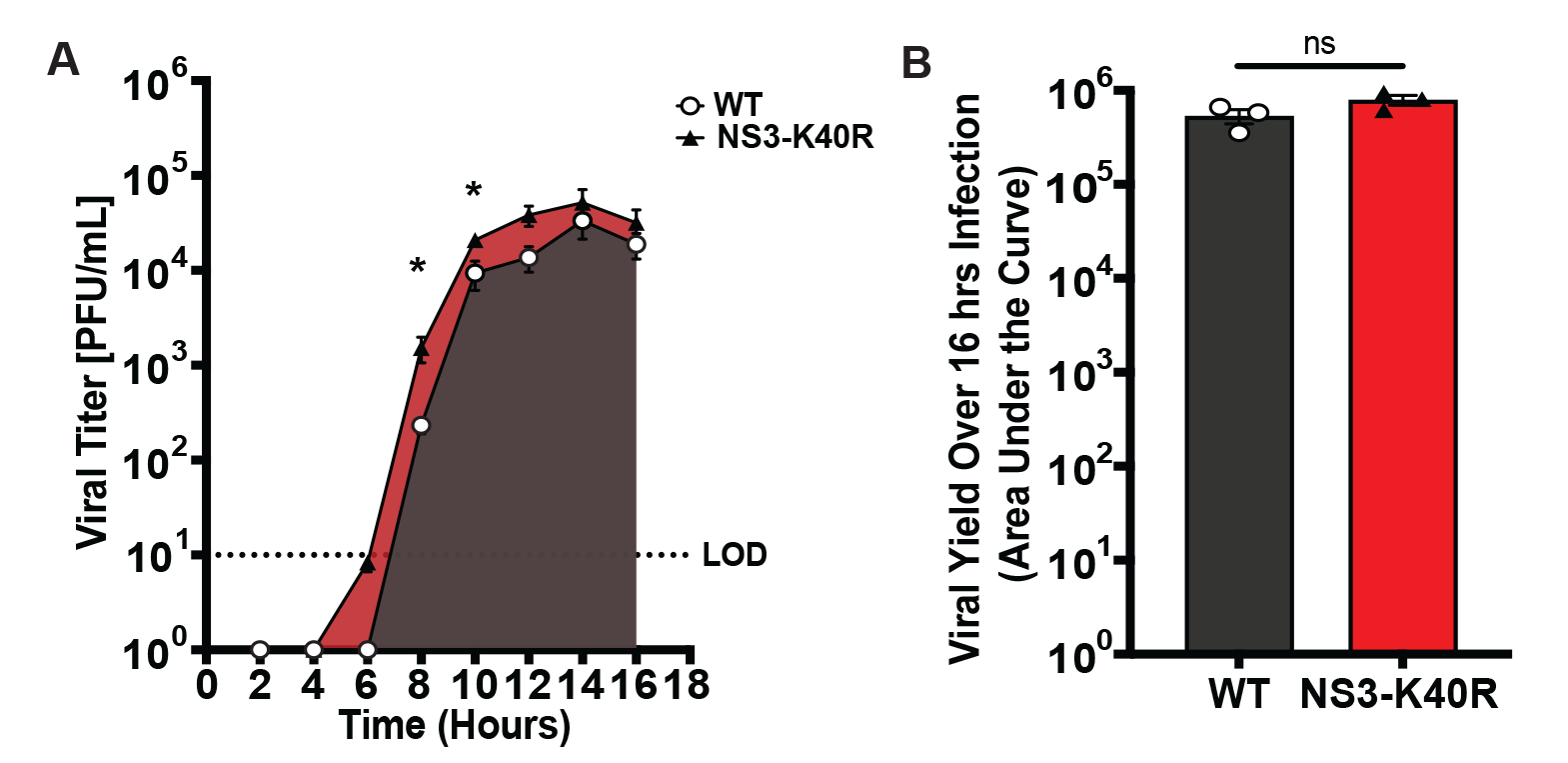

Supplement: S1 Fig — (A) Single-cycle growth curve of WT and NS3-K40R extracellular viruses. BV2 cells were infected at MOI 0.1 and supernatant was collected and viral titer determined via plaque assay. Samples without detectable plaques are displayed as 1 (below the limit of detection, LOD) for visualization purposes. Data are mean ± SEM (n = 3, ≥ 3 independent experiments). *P < 0.05, unpaired t test. (B) Total viral load is similar between WT and NS3-K40R. The area under the curves in panel A are displayed. Data are mean ± SEM (n = 3, ≥ 3 independent experiments). The data underlying this figure can be found in S1 Data file, Tabs 22–23. (TIF) [file pbio.3003245.s001.tif]

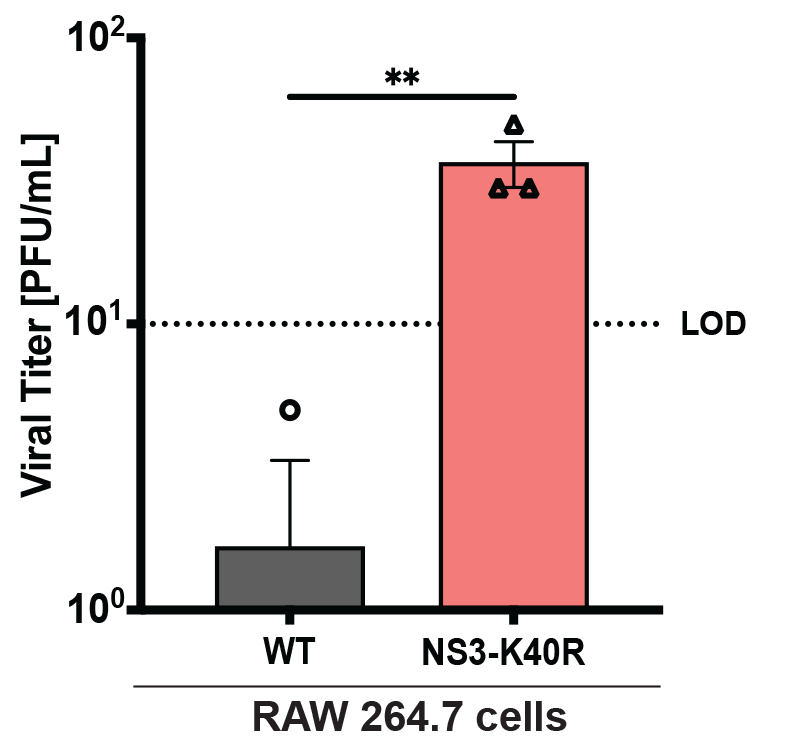

Supplement: S2 Fig — RAW264.7 cells were infected with WT and NS3-K40R at MOI 0.1 for 8 h. Extracellular virus was measured via plaque assay. Data are mean ± SEM (n = 3, ≥ 3 independent experiments). *P < 0.05, unpaired t test. LOD, limit of detection. The data underlying this figure can be found in S1 Data file, Tab 24. (TIF) [file pbio.3003245.s002.tif]

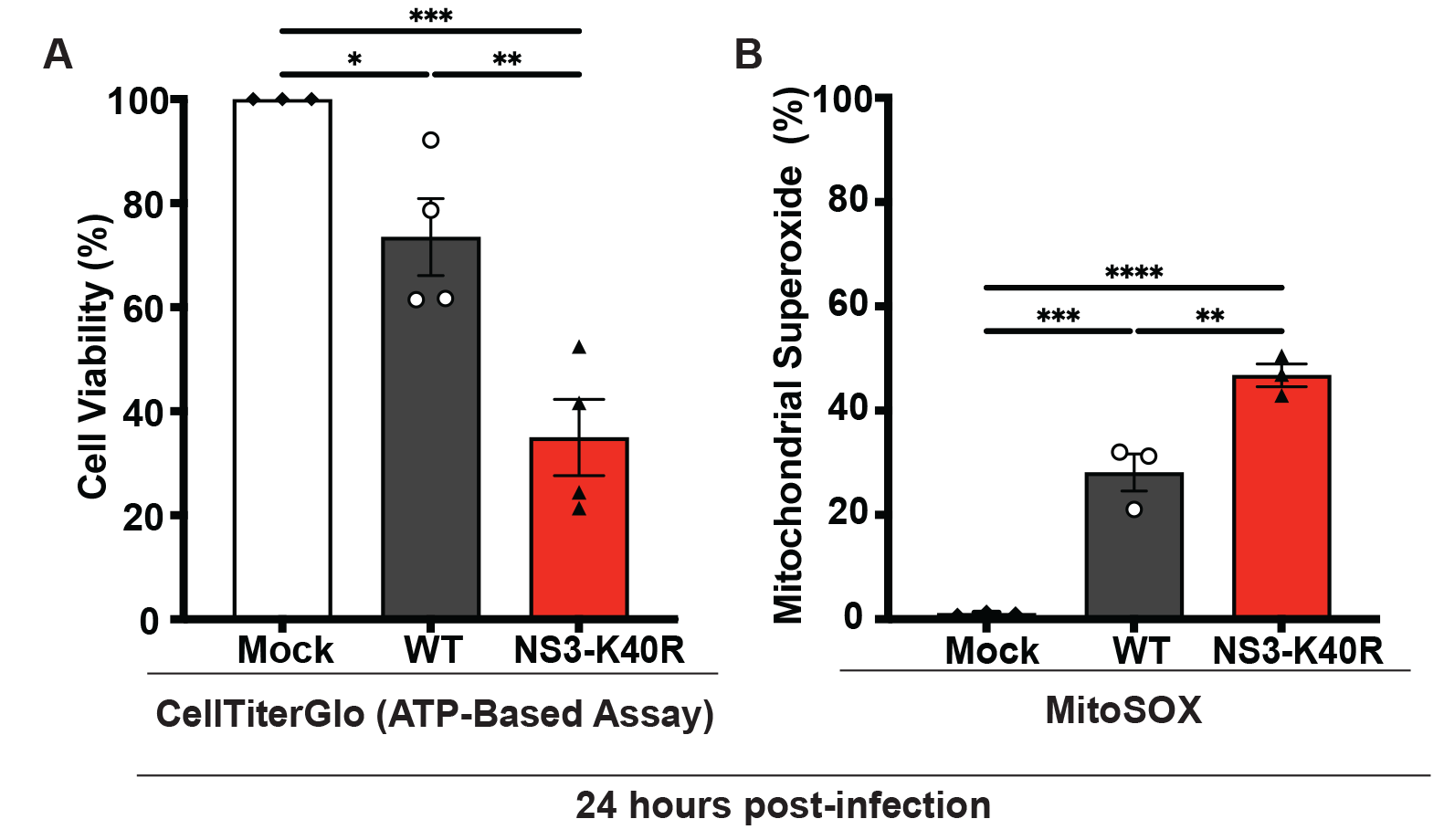

Supplement: S3 Fig — (A) NS3-K40R increases cell death at 24 h post-infection. BV2 cells were infected with mock, WT or NS3-K40R at MOI 2 and at 24 h post-infection cell viability was quantified using ATP-based assay. Data are mean ± SEM (n = 4, ≥ 3 independent experiments). *P < 0.05, one-way ANOVA. (B) NS3-K40R increases mitochondrial superoxide at 24 h post-infection. BV2 cells were infected with mock, WT or NS3-K40R at MOI 2 and at 24 h post-infection mitochondrial superoxide was quantified by flow cytometry to detect MitoSOX staining. Data are mean ± SEM (n = 3, ≥ 3 independent experiments). *P < 0.05, one-way ANOVA. The data underlying this figure can be found in S1 Data file, Tabs 25–26. (TIF) [file pbio.3003245.s003.tif]

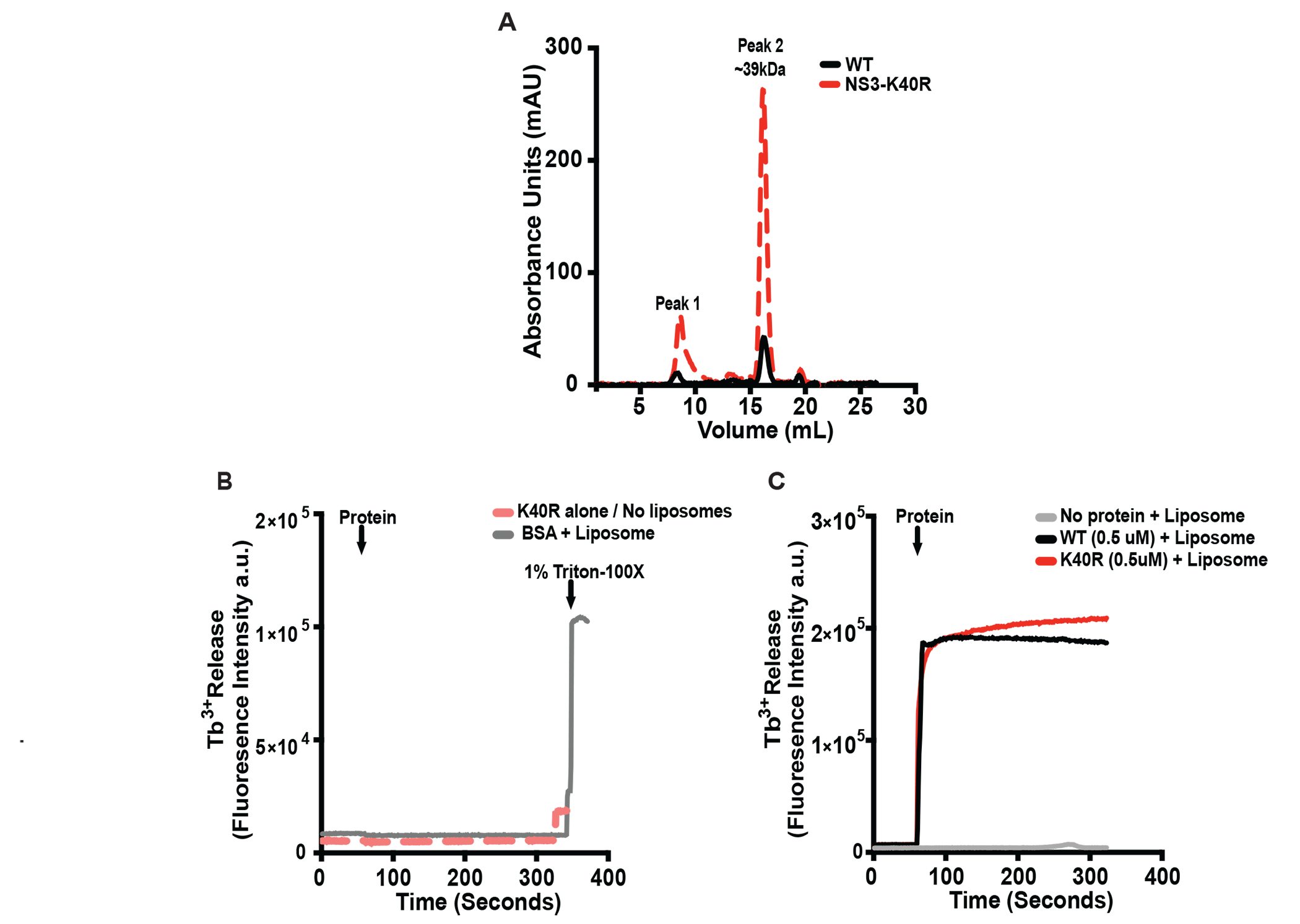

Supplement: S4 Fig — (A) Purification of tagless NS3 proteins for liposome leakage assays. Purified His6-MBP-NS3 fusion proteins were treated with TEV protease to remove the His6-MBP tag. Following cleavage, the proteins were purified using Ni-NTA resin and subjected to size-exclusion chromatography. Absorbance peaks from size-exclusion chromatography are shown (NS3-WT in black and NS3-K40R in red) and the peak at approximately 16–17 mL was collected for use in liposome leakage assays. (B and C) Controls for the liposome leakage assay. (B) NS3-K40R was incubated in the absence of liposomes, or 0.5 µM BSA was incubated with cardiolipin liposomes (0.1 mM; 80% phosphatidylcholine and 20% cardiolipin) and at the end of the experiment detergent was added to disrupt liposomes. The leakage of Tb³+ fluorescence leakage was quantified upon binding to dipicolinic acid (DPA). Data are representative of two independent experiments. (C) Experiment was repeated as in B, but cardiolipin liposomes (0.1 mM; 80% phosphatidylcholine and 20% cardiolipin) were incubated with purified NS3-WT and NS3-K40R proteins at 0.5 µM or no protein, and leakage of Tb³+ fluorescence leakage was quantified upon binding to DPA. Data are representative of two independent experiments with separate protein/liposome preparations. a.u., arbitrary units. The data underlying this figure can be found in S1 Data file, Tabs 27–29. (TIF) [file pbio.3003245.s004.tif]

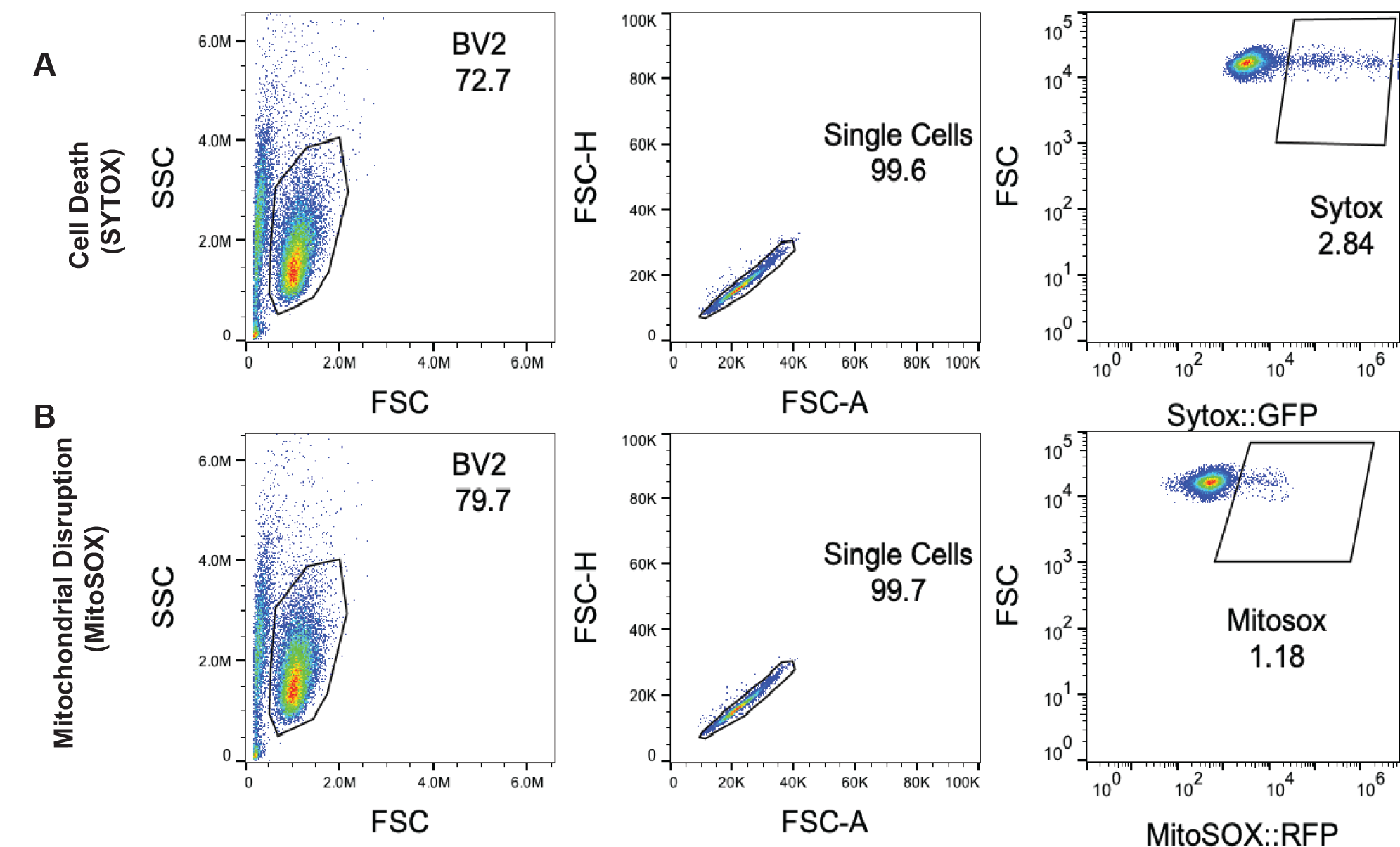

Supplement: S5 Fig — Representative flow cytometry plots showing the gating strategy used to measure (A) cell death and (B) mitochondrial disruption. BV2 cells were first gated based on forward and side scatter to exclude debris and select viable cell populations. Single cells were then identified using FSC-H vs. FSC-A gating. Within the single cell population, SYTOX-positive cells were gated to quantify cell death (A), or MitoSOX-positive cells were gated to assess mitochondrial reactive oxygen species production (B). This gating strategy was applied to the data shown in Fig 5A–5D. (TIF) [file pbio.3003245.s005.tif]

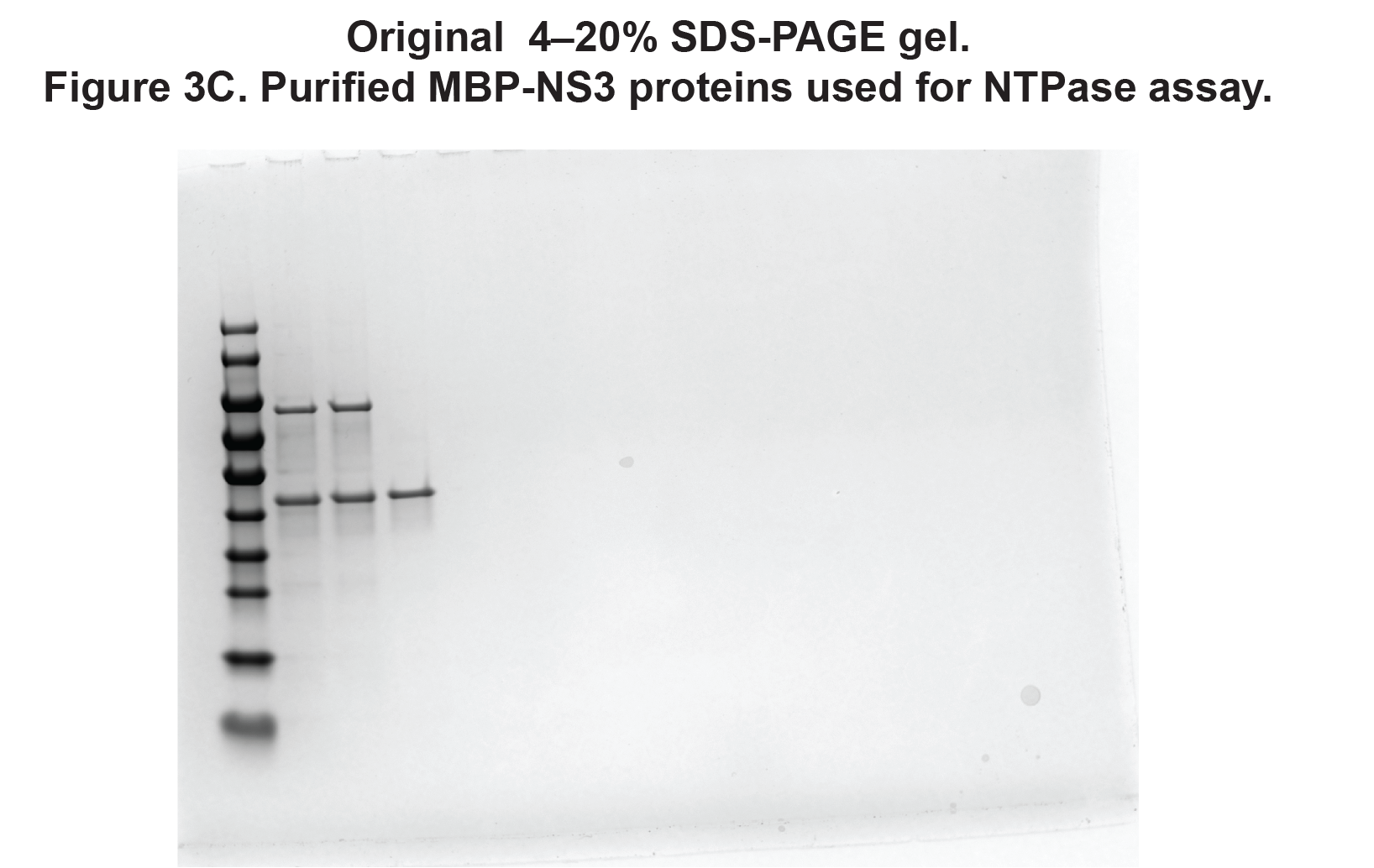

Supplement: S1 Raw Image — Purified MBP-NS3 proteins used for NTPase assay. MBP-NS3 fusion proteins or MBP protein alone were purified and analyzed by 4%–20% SDS-PAGE, followed by Coomassie Blue staining. The bands of proteins were quantified using ImageJ. (TIF) [file pbio.3003245.s007.tif]
