# Supplementary material for: Egress-enhancing mutation reveals the inefficiency of non-enveloped virus cell exit
Source: PLoS Biol. 2025 Jun 24;23(6):e3003245. doi: 10.1371/journal.pbio.3003245 (PMC12212872; doi:10.1371/journal.pbio.3003245)
Supplement: Attachment — Submitted filename: VRI final rebuttal.docx [file pbio.3003245.s008.docx]

We thank the reviewers for their valuable feedback and thoughtful comments on this manuscript. Each comment has been addressed through new experiments (infection data from immune-competent mice, quantification of total viral yields over a single cycle, quantification of vRNA:PFU ratios), clarification of data displays (showing primary flow cytometry data and non-normalized data for cell death and mitochondrial disruption assays), and/or text changes as shown in blue responses below.

Reviewer #1: The studies by Rodriguez-Irizarry et al. isolate and characterize a novel adaptive mutation in murine norovirus that enables increased early replication. They convincingly prove that the functional adaptive mutation is within NS3 and characterize molecular functions of purified WT and mutant NS3. They propose that the mechanism of increased early replication is due to increased mitochondrial disruption, cell death, and virion release during early infection. The study is well conceived, and most data is presented clearly with thoughtful discussion. My main critique is related to mechanistic uncertainty about how early death would translate to increased virion production. Specific points of critique follow Major points

1. The major point of confusion surrounds how early disruption of mitochondria would relate to more PFU produced. It doesn't make sense to me that earlier death and more extracellular PFU would result in greater total PFU but should just shift the proportion of intracellular/extracellular. Does this imply that virion assembly is regulated by cell death? One way this may be regulated is through more rapid production of encapsidated virions. The authors could quantify VP1 by western blot to determine whether there is earlier protein production.

Based on our original data we hypothesize that, as the reviewer suggests, the NS3-K40R mutation does not alter the amount of virus produced—it simply alters the timing of release of the virus, with progeny detectable earlier than WT virus. If this is the case, then WT and NS3-K40R viruses should have equivalent total yields over a full single cycle of infection. To formally test this possibility, we performed a new experiment (new Supplemental Figure 1) where we infected cells with WT or NS3-K40R and collected and quantified extracellular virus every 2 h over an entire 16 h single cycle infection. In agreement with all of our other data, NS3-K40R has increased yields at early timepoints (6-10 hpi), but by the end of the time course WT virus “catches up” (Supp. Fig. 1A). Comparing the area under the curves revealed that total viral yields for WT and NS3-K40R are equivalent (Supp. Fig. 1B). Combined with other new data (discussed below), this new experiment reveals insights into a question raised by multiple reviewers: Why is lysine maintained at position 40 in MNV populations if arginine promotes earlier viral release? At least part of the answer may be that WT and NS3-K40R virus yields are equivalent over an entire single cycle.

These new data are shown in new Supplemental Figure 1, and are described in the text in lines 219-228:

*“To gain finer insight into egress kinetics, we performed additional infection experiments where BV2 cells were infected at an MOI 0.1 and extracellular viral progeny in the media were collected every two hours over a 16-hour period. We found that at early time points, between 6 and 10 hours post-infection, the NS3-K40R had higher viral titers than WT (Supplemental Figure 1A). However, at later time points, no significant differences were observed between WT and NS3-K40R. To determine the total viral yields for WT and NS3-K40R over the 16 hour infection, we calculated the area under the curve and found no significant differences (Supplemental Figure 1B). These data suggest that while NS3-K40R may confer an early advantage in viral replication, the overall viral yield across the infection time course is similar for NS3-K40R and WT.”*

2. Related to the prior point, is there a difference in genome:PFU ratio? Figure 3A shows no difference in viral genomes at the same time points where there is a difference in PFU shown in prior figures. Presumably these genomes are exclusively from the cell associated fraction, but this should be clearly stated in the legend and/or methods. In addition to clarity on the source of genomes in Fig. 3A, it would be informative to quantify genome:PFU ratio from cell and supernatant for WT and mutant viruses to determine whether there is a qualitative difference in the PFU produced.

As suggested by the reviewer, we now state more clearly that our RNA quantification in Figure 3A was exclusively from cells rather than including extracellular samples. As discussed in point 1 above, intracellular RNA levels for WT and NS3-K40R are equivalent, but NS3-K40R virions are released earlier. Text changes clarifying RNA quantification are included in the Figure 3A legend, “*BV2 cells were infected with WT or NS3-K40R at MOI 2, and intracellular viral RNA at indicated time points was harvested and quantified via RT-qPCR targeting MNV ORF1”* (Line 179-180) and Methods *“At 2, 4, and 6 hrs post-infection, cells were collected, and intracellular RNA was isolated using TRIzol”* (Line 505-506).

To address the reviewer’s question about potential changes in genome:PFU ratios, we performed a new experiment and found no differences in the vRNA:PFU ratio of WT and NS3-K40R, now included as new Figure 3B and are described in the text in lines 170-177:

*“Second, we compared the replication capacity of WT and NS3-K40R by calculating the vRNA:PFU ratio. BV2 cells were infected at MOI 2, and cells were collected 10 hours post-infection. Half of the cell volume was used for qRT-PCR to quantify genome copies as described above, while the other half was used for plaque assays to determine viral titers. We then calculated the vRNA:PFU ratio by dividing the number of genome copies by the viral titer. No significant differences in the vRNA:PFU ratio were observed (Figure 3B), indicating that NS3-K40R does not alter vRNA or virion production at 10 hours post-infection.”*

3. In Figure 4, there appears to be less PFU in the extracellular virus fraction from each virus strain than from the corresponding cell-associated fraction. This is puzzling because the earlier phenotype of the combined fractions in figure 1 would be expected to most reflect the majority of PFU present in the cell-associated fraction. Is this due to technical considerations related to tittering only a portion of the extracellular fraction or can this be explained in some other way?

We understand the confusion, and the reviewer is correct—early in infection extracellular titers are lower than intracellular titers (Figure 4), but in Figure 1 we observe increased yields for P11 viruses at early time points from pooled extracellular + intracellular progeny. We think that this discrepancy is due to differences in the viruses for these two figures: Figure 1 analyzes a heterogeneous, passaged viral population containing a mixture of faster-replicating variants (P11-6 hrs) including but not limited to NS3-K40R, whereas Figure 4 analyzes a cloned virus stock containing only the NS3-K40R mutation. Thus, differences in total viral yields and kinetics between Figure 1 and Figure 4 are likely influenced by differences between passaged versus cloned viral populations.

We address this point in the text in lines 448-453:

*“Note that other minor variants may be present in P11-6 hr samples that were not abundant enough to detect by consensus sequencing, but may contribute to phenotypes shown in Figure 1. The Passage 11-24 hr samples also contained the NS3-I340T mutation, but not the NS3-K40R or silent mutation in VP2. It is possible that the NS3-I340T mutation, in combination with NS3-K40R, contributes to increased replication speed of the P11-6 hr viral population (Fig. 1C), but this was not evaluated here.”*

4. Text describing figure 3C states that there is no difference in enzymatic activity of WT/K40R. However, the data seem insufficient to support this claim because Fig. 3C data show an extent of activity, not a rate. The claim in the text should be revised, or kinetics curves should be furnished.

The reviewer is correct—we did not perform kinetic analysis so we have softened our conclusion in the text in line 201-203 ‘*However, no apparent differences in ATP hydrolysis were observed between WT and K40R after 30 minutes of reaction.’*

5. Investigators comment in the text that an amino acid 1-20 deletion of NS3 abrogates mitochondrial localization. Does the K40R mutation still have an effect in the context of a 1-20aa deletion? This may be beyond the scope if the current study but would go some way to strengthening the mechanistic model of mitochondrial involvement.

The importance of the first 20 amino acids in NS3 for mitochondrial localization was previously demonstrated by Wang et al.—deletion of the N-terminal 20 amino acids renders MNV non-infectious due to lack of cell death and viral egress. Interestingly, this mutant virus has unimpaired RNA replication, etc., and manual release of progeny by freeze-thaw lysis generates viruses capable of infecting naïve cells and undergoing RNA replication. In the current study, we focused on characterizing the phenotype of the NS3-K40R mutation in the context of full-length NS3. While we did not directly test the effect of NS3-K40R in the context of an N-terminal (1–20 amino acid) deletion, we speculate that deletion of the N-terminal 20 amino acids would prevent cell death and egress regardless of any other NS3 mutations (including K40R). In other words, K40R would not overcome the severe defect of NS3Δ20 mutants. We add more discussion of these topics in the text in lines 376-380:

*“For NS3, Wang et al. suggest that the mitochondrial localization signal in amino acids 1-20 of NS3 directs the protein to mitochondria followed by membrane disruption via four-helix bundle domain in amino acids 25-158. Deletion of the N-terminal 20 amino acids of NS3 prevented MNV-mediated cell death and egress, thus blocking detectable viral spread in vitro and in vivo [31].”*

6. Are titers of K40R virus different from WT during persistent infection of a non-immunodeficient mouse? This may be beyond the scope but would substantially increase the authors ability to discuss the evolutionary advantage or lack thereof.

To address this, we performed new experiments where we infected wild-type immunocompetent mice with WT or NS3-K40R viruses and examined viral titers at 24 hours post-infection in the ileum and colon (new Figure 6G-H). Interestingly, we found no significant differences in viral titers in WT vs. NS3-K40R infected immune-competent animals. Therefore, although NS3-K40R has higher titers than WT in immune deficient *Stat1^-/-^* mice, titers in immune-competent animals are equivalent. These data, along with new data showing equivalent yields for both viruses over an entire 16 h infection course in vitro, aid our understanding of why the WT lysine is maintained at position 40 of NS3—NS3-K40R viruses have enhanced egress early, but WT virus “catches up” with equivalent total yields. Higher titers of NS3-K40R in *Stat1^-/-^* mice but not wild-type mice is interesting and may suggest that in immune-competent animals, STAT1-dependent innate responses are triggered sooner by early viral egress of NS3-K40R virions, thus limiting the advantage of NS3-K40R.

New mouse data is shown in Figure 6G-H and is described in the Results in lines 317-324 and in the Discussion in lines 406-411:

*“Second, we repeated the experiment in Stat1^-/-^ mice as well as in immunocompetent mice but sacrificed mice at 24 hours post-infection for titer analysis via plaque assays [41]. NS3-K40R-infected Stat1^-/-^ mice had increased viral titers in the spleen, duodenum, ileum and colon (Figure 6C-F). However, we did not observe differences in WT vs. NS3-K40R viral titers from immunocompetent mice (Figure 6G-H). Overall, the increased titers of NS3-K40R in Stat1^-/-^ mice suggest that this mutation enhances viral replication early in infection within an immunocompromised host, but this advantage is lost in mice with intact innate immune responses.”*

*“Another possibility is that early release of progeny induces early host immune responses that would then limit replication. Importantly, although NS3-K40R has higher titers than WT in Stat1^-/-^ mice, this titer difference is lost immune-competent animals (Figure 6C-H). Therefore, it is possible that in immune-competent animals, STAT1-dependent innate responses are triggered sooner by early viral egress of NS3-K40R virions, thus limiting the advantage of NS3-K40R.”*

Minor points

1. Please show representative sytox and mitoSOX flow cytometry data.

The representative data from flow cytometry have been added as Figure 5A and 5C.

2. In figures 5A-B and S3B-C, the y-axis scales vary substantially. On the face of it, this does not present a problem, but the amount of release in S3C stimulated by 0.5 uM K40R is ~1/3 that shown to be stimulated by 0.05 uM K40R in 5C. Were these experiments scaled to one another to compare max signal for each replicate? If so, can the investigators show these data? If not, can the investigators comment on how to reconcile these variations in leakage magnitude?

We did not scale or normalize the experiments in panels mentioned by the reviewer (now Fig. 5E-F and Supp. Fig. 3B-C) to directly compare the maximum signal for each replicate. The observed differences in leakage magnitude are likely due to the fact that we use freshly purified viral proteins for these assays (within 3 days of size-exclusion chromatography) and different liposome preparations. Specifically, Figures 5E-F were generated using one liposome preparation, whereas the data in Supplemental Figure S3C were derived from an independent liposome preparation.

We addressed these points in our methods section in lines 584-593:

*“For liposome leakage assays, 0.1 mM of Tb^3+^-encapsulated liposomes were suspended in 250 μl buffer C (20 mM HEPES (pH 7.5) 150 mM NaCl and 50 μM of DPA) and NS3 or control proteins were added. Due to the sensitivity of these assays, NS3 proteins were used within 3 days of size-exclusion chromatography. Fluorescence of Tb^3+^ was monitored over time on a PTI spectrofluorometer using λ_Ex_=490 nm and λ_Em_=276 nm. At the end of the incubation, 1% Triton X-100 was added to measure complete release of Tb^3+^. Liposome preparations can vary leading to discrepancies in overall signal intensity. Therefore, comparisons were made only between assays using a given liposome preparation. Figure 5E-F shows analyses performed with a single, shared liposome batch, whereas the data in Supplemental Figure 4 were generated using an independent liposome preparation.”*

3. Units for figures 3C, 5A, 5B are difficult to interpret. 3C appears to represent a concentration, of inorganic phosphate released from ATP hydrolysis, presumably calculated from a standard curve of absorbance-based measurements; would be better presented as raw absorbance with standard curve or as simply "free Pi", eg. 5A and B are described as being the difference between % of infected and mock cells in methods but as simply "normalized to uninfected" in figures.

We have made the suggested adjustments to the figure legends and graphs to improve clarity.

For Figure 3C (now Fig. 3D), we have updated the y-axis label to ‘*Inorganic Phosphate (µM)*’. In our Methods section, we now clarify how these values were calculated: *‘A standard curve was used to determine the linear concentration of inorganic phosphate released during the reaction.’*

For Figure 5, there have been several improvements based on the reviewer’s suggestions. First, we now show the primary flow cytometry data (now Fig. 5A/C). Second, for Figs. 5B/D (formerly Fig. 5A/B), we removed our previous depiction of normalized data (to uninfected) and instead now show the percentage of positive cells for mock vs. WT vs. NS3-K40R. These changes aid visualization of the data and are parallel with the primary flow cytometry data.

4. Several cases of data below the LOD in titer assays - figures 6c, 4b, 2b, S1. How is LOD determined in these cases? Are statistics performed on values below the LOD?

We established our LOD for our in vitro experiments at 2 plaques in the undiluted samples. In Figure 6, the LOD was based on tissue weight and a threshold of 3 plaques in the undiluted samples. All values above zero were considered for statistical analysis: For example, an undiluted tissue sample that yielded 2 plaques would generate a data point below the LOD, but it would be shown on the graph and used in the statistical comparison.

We have clarified this in our methods section in Line 490-492 and 601-604:

*‘We set the limit of detection (LOD) at 2 plaques in undiluted samples (10 PFU); therefore, in some cases, values were below this detection limit. Samples with 1 or more plaques were included in the statistical analysis.’*

*‘We set the limit of detection (LOD) at 3 plaques in undiluted samples and considered the average tissue weight to calculate the PFU/grams tissues; therefore, in some cases, values were below this detection limit. Samples with 1 or more plaques were included in the statistical analysis.’*

------------------------------------

Reviewer #2: This paper is well written, with a straight forward experimental design. It seeks to identify how murine norovirus may adapt to selective pressure requiring faster infection, replication or production rates. The authors passage and collect virus at 6 hours versus 24 hours across 11 passages. They identify two amino acid changes in NS3, one of which is unique to the 6 hour early collection timepoint. The paper confirms that this mutation results in faster virus replication, virus production and egress, both in cell culture and in vivo. I have no critical concerns wth the experiments as they are performed and am convinced by the data. There are two points I wish to raise that could be considered for additional experimentation, without which the study seem incomplete. However, I don't think it absolutely essential to perform. These studies would not negate the current results, but they would possibly provide a more complete picture.

One, is the fact that the I340T mutation was not studied, because it appeared in both 6 and 24 hour timepoints and thus considered to be cell adaptation unrelated to early passage. However, one could imagine that it still somehow confers this similar increase in replication and egress, and that it simply emerges at the later time-point. There is also question of how the double mutant would behave. The discussion does mention that they observed a mutation at this position in another cell line (this time it was I340V, but I do not believe the phenotype of this mutation was studied there either. Of course this would ask for more experimental work and might confuse the intended narrative, but could be considered by the authors. Perhaps they have already tried.

Regarding the I340T mutation, we have not tested the phenotype as a single or double mutation with NS3-K40R. We speculate that it could have an effect on the replication of MNV3; however, it may or may not be associated with the enhanced replication speed that we aimed to investigate in this study given that it also arose in the non-selective condition (24h passaging). In a previous unrelated study in our lab, we also identified a mutation at NS3-340, in this case I340V rather than the I340T mutation observed here. This previous study was focused on adapting MNV to a human cell line. Therefore, it is likely that mutation at NS3 position 340 is a common adaptation to cell culture replication. That said, we cannot rule out possible contributions of I340T to our enhanced egress phenotype, particularly given subtle differences in our bulk population assays (Fig. 1) vs. NS3-K40R cloned virus assays (Fig. 4)—see our answer to Comment 3 from reviewer 1 above.

We have clarified these points in the manuscript in the Discussion and Methods in Lines 117-123 and 450-455.

*“The mutation at nucleotide 2047 resulted in an amino acid substitution from isoleucine to threonine (I340T) in non-structural protein 3 (NS3), which was present in both Passage 11-6 hrs and Passage 11-24 hrs. Given that I340T was present in both selective (Passage 11-6 hrs) and non-selective (Passage 11-24 hrs) conditions, it is possible that this mutation is due to drift or general cell culture adaptation. Indeed, we recently identified a mutation at this site, I340V, in MNV genomes following adaptation to replication in HeLa cells [35]. Both mutations found at I340 appear to be associated with general cell culture adaptation.”*

*“Note that other minor variants may be present in P11-6 hr samples that were not abundant enough to detect by consensus sequencing, but may contribute to phenotypes shown in Figure 1. The Passage 11-24 hr samples also contained the NS3-I340T mutation, but not the NS3-K40R or silent mutation in VP2. It is possible that the NS3-I340T mutation, in combination with NS3-K40R, contributes to increased replication speed of the P11-6 hr viral population (Fig. 1C), but this was not evaluated here.”*

The other question is whether the authors could perform a direct competition assay in vivo (better) or in vitro, that might indicate that while this mutation does improve egress and virus production, it might not so easily outcompete wildtype virus, and thus, it only emerges when selective pressure leans heavily in its favor. These can both be experimentally addressed, or discussed.

As the reviewer suggests, we also thought about performing serial passage fitness competition experiments to examine potential fitness costs of the NS3-K40R mutation (as we have done in the past—we like these approaches: Lanahan PNAS 2021, Robinson CHM 2014, Pfeiffer PLoS Path 2005). However, the phenotype here—cell death and egress—creates unique caveats for these sorts of experiments. Any cell co-infected by both WT and NS3-K40R will release both viruses at the same time, even if it was K40R’s NS3 protein that was responsible for early cell death and viral egress. Therefore, co-infection would render these experiments uninterpretable for addressing the desired questions. It may be possible to control for these effects in vitro if passages are done at low MOI with titer assays in between each cycle to ensure continued low MOI in each passage (possible, but labor-intensive). However, in mice, we can’t control for co-infection.

Instead, we think that our new data from immunocompetent mice goes a long way to address the core of the reviewer’s question—does NS3-K40R really have a fitness advantage over WT in physiological contexts where the virus evolved? The answer is no—see our response to reviewer 1 comment 6.

------------------------------------

Reviewer #3: This manuscript investigates the replication efficiency of murine norovirus (MNV), a non-enveloped virus, focusing on the viral egress stage. The central finding is the identification and characterization of a single amino acid mutation in the MNV non-structural protein 3 (NS3), specifically a lysine to arginine change at position 40 (K40R), which significantly enhances viral egress and overall replication speed. The study employs a forward genetic approach to uncover this previously masked inefficiency in the viral life cycle. Rodriguez-Irizarry and colleagues used a forward genetic screen involving sequential harvesting of early viral progeny (at 6 hours post-infection) to enrich for faster-replicating MNV mutants in cultured BV2 cells. After 11 passages, they identified a virus (Passage 11-6 hrs) with significantly higher yields at early time points compared to the wild-type virus (Passage 0). As shown in Figure 1B, for the comparison between Passage 0 and Passage 11-6 hrs at 6 hours post-infection. Figure 1C also demonstrates for higher viral titers of Passage 11-6 hrs at early time points in a single-cycle growth curve. Sequencing of the fast-replicating virus revealed three mutations, one of which, a missense mutation resulting in the K40R substitution in the NS3 protein, was identified as crucial for the enhanced phenotype. Further experiments using infectious clones with the individual mutations confirmed that NS3-K40R was sufficient to confer the faster replication phenotype. Figure 2B shows that the "NS3-K40R" mutant displayed significantly higher viral yields compared to the wild-type (WT) virus. The study found that the NS3-K40R mutant induced earlier cell death compared to the wild-type virus. Figure 5A shows a significant increase in Sytox-positive dead cells at 6 and 8 hours post-infection for the NS3-K40R virus. The enhanced cell death correlated with increased mitochondrial dysfunction, as evidenced by elevated levels of mitochondrial superoxide production at early time points in cells infected with the NS3-K40R mutant. Mechanistically, the NS3-K40R protein exhibited a greater ability to disrupt cardiolipin-containing membranes in vitro compared to the wild-type NS3 protein, particularly at lower protein concentrations (Figure 5C). This suggests that the mutation enhances the membrane-disrupting activity of NS3, potentially leading to mitochondrial damage and cell death. The NS3-K40R mutation did not significantly alter viral RNA synthesis or the NTPase activity of the NS3 protein. Figure 3A shows no significant difference in viral RNA levels between WT and NS3-K40R at most time points. Figure 3C demonstrates that "no differences in ATP hydrolysis were observed between WT and K40R". This indicates that the enhanced replication is likely due to improved egress rather than increased RNA production. Infection of Stat1-/- mice (immunocompromised mice lacking the type I interferon receptor signaling) with the NS3-K40R virus resulted in increased viral titers in various tissues (spleen, duodenum, ileum, and colon) at 24 hours post-infection (Figure 6 C-F, P<0.05 for several tissue comparisons). This suggests that the egress-enhancing mutation does not reduce viral fitness in vivo in the absence of a robust interferon response. While the K40 residue in NS3 is generally conserved as lysine among murine noroviruses, some caliciviruses have arginine at this position. The study raises interesting questions about the selective pressures that maintain lysine at this position in MNV, suggesting potential fitness costs associated with enhanced egress that were not observed in this study (e.g., impact on host immune response or interhost transmission). This study provides novel insights into the replication strategy of non-enveloped viruses by revealing a previously unrecognized inefficiency in the egress process of MNV. In addition, the identification and characterization of the NS3-K40R mutation highlight the crucial role of NS3 in viral egress and its link to host cell death and membrane disruption. The findings have implications for understanding the selective pressures balance that shape viral evolution and fitness. While the effects are subtle, the data strongly support the thoughtful and well-written interpretation of the results.

The study presents compelling findings, but one notable shortcoming is the lack of direct evidence demonstrating that the NS3 K40 mutant is faster specifically because it accelerates cell death. The observed effect on mitochondrial function remains correlational, leaving room for alternative explanations. For example, it's possible that the increased speed of the mutant could be a secondary consequence of a virus replication function that wasn't fully explored or considered within this study. Further analysis of this potential mechanism could strengthen the conclusions.

Regarding the link between NS3 and cell death/mitochondria function we agree with the reviewer that the precise mechanism by which NS3-K40R differentially mediates cell death through mitochondria is unclear. Although we did not establish a direct connection between mitochondrial dysfunction and cell death, it would be interesting for future studies to explore the precise mechanism by which NS3 mediates cell death via mitochondrial disruption, through structural studies, for example.

We clarified this point in the manuscript in Line 392-394: *‘Although the precise mechanism by which NS3-K40R disrupts mitochondria remains unknown, these results provide new insight into NS3-mediated viral egress.’*

Additionally, the evolutionary implications of these findings are intriguing. If the NS3 K40 mutant competes with the wild type (WT) over multiple passages, it raises the question: which variant is ultimately fitter? This could be addressed with targeted experiments in cell culture, but ideally, it would also be tested in animal models to provide a more comprehensive understanding. The central question remains why this mutant, despite showing higher titers in both cell culture and animal studies, has not emerged as a dominant variant through evolutionary processes. Could there be unknown selective pressures or trade-offs that prevent the mutant from outcompeting the WT in natural settings? Exploring these aspects would provide valuable insights into viral evolution and the dynamics of fitness trade-offs in infection and transmission.

The reviewer raises a great point, also highlighted by the other reviewers as well. Our new experiments resolve many of these questions. Although NS3-K40R has higher titers than WT in immune deficient *Stat1^-/-^* mice, their titers in immune-competent animals are equivalent (new data in Figure 6GH). These data, along with new data showing equivalent yields for both viruses over an entire 16 h infection course in vitro (new Supplemental Figure 1), aid our understanding of why the WT lysine is maintained at position 40 of NS3—NS3-K40R viruses have enhanced egress early, but WT virus “catches up” with equivalent total yields. Higher titers of NS3-K40R in *Stat1^-/-^* mice but not wild-type mice is interesting and may suggest that in immune-competent animals, STAT1-dependent innate responses are triggered sooner by early viral egress of NS3-K40R virions, thus limiting the advantage of NS3-K40R. For more detailed information, see our responses to reviewer 1 comments 1 and 6.
